# Neuroimaging evaluation of the long term impact of a novel paired meditation practice on brain function

**DOI:** 10.3389/fnimg.2024.1368537

**Published:** 2024-06-10

**Authors:** Andrew B. Newberg, Nancy A. Wintering, Chloe Hriso, Faezeh Vedaei, Sara Gottfried, Reneita Ross

**Affiliations:** ^1^Department of Integrative Medicine and Nutritional Sciences, Thomas Jefferson University, Philadelphia, PA, United States; ^2^Department of Radiology, Thomas Jefferson University, Philadelphia, PA, United States; ^3^Department of Obstetrics and Gynecology, Thomas Jefferson University, Philadelphia, PA, United States

**Keywords:** cerebral metabolism, positron emission tomography (PET), meditation, brain imaging, sexual

## Abstract

**Background:**

A growing number of advanced neuroimaging studies have compared brain structure and function in long term meditators to non-meditators. The goal is to determine if there may be long term effects on the brain from practicing meditation. In this paper, we present new data on the long term effects of a novel meditation practice in which the focus is on clitoral stimulation. The findings from such a study have implications for potential therapeutic uses with regard to various neurological or psychiatric conditions.

**Methods:**

We evaluated the cerebral glucose metabolism in 40 subjects with an extended history (>1 year of practice, 2–3 times per week) performing the meditation practice called Orgasmic Meditation (OM) and compared their brains to a group of non-meditating healthy controls (*N* = 19). Both meditation and non-meditation subjects underwent brain PET after injection with 148 to 296 MBq of FDG using a standard imaging protocol. Resting FDG PET scans of the OM group were compared to the resting scans of healthy, non-meditating, controls using statistical parametric mapping.

**Results:**

The OM group showed significant differences in metabolic activity at rest compared to the controls. Specifically, there was significantly lower metabolism in select areas of the frontal, temporal, and parietal lobes, as well as the anterior cingulate, insula, and thalamus, in the OM group compared to the controls. In addition, there were notable distinctions between the males and females with the females demonstrating significantly lower metabolism in the thalamus and insula.

**Conclusions:**

Overall, these findings suggest that the long term meditation practitioners of OM have different patterns of resting brain metabolism. Since these areas of the brain in which OM practitioners differ from controls are involved in cognition, attention, and emotional regulation, such findings have implications for understanding how this meditation practice might affect practitioners over long periods of time.

## Introduction

Neuroimaging studies have been widely used in the study of various mind-body practices such as meditation, prayer, or yoga, to elucidate neurophysiological correlates. Such data has helped gain an understanding of the brain mechanisms associated with these practices as well as explore potential clinical effects. A number of neuroimaging studies have reported structural and functional differences in the brain of long-term meditators compared to non-meditators (Luders and Kurth, 2019). These findings have provided a greater understanding of the brain structures and functional networks involved in various meditative practices, particularly when done over extended periods of time. Brain regions associated with meditative practices have included parts of the frontal and parietal lobes, limbic structures, and larger networks such as the salience network and default mode network (DMN). Other studies have demonstrated that various distinct meditation programs can affect cognition, emotion, and other brain processes over longer periods of time. Such findings are related to, but in contrast to brain changes that occur during the meditation practice itself (Afonso et al., 2020). For example, several studies have documented that meditation practice can be associated with changes in how the brain responds to various emotional or cognitive tasks (Manna et al., 2010; Travis, 2014; Zhang et al., 2019; Lin et al., 2021; Whitfield et al., 2022). Several different studies have also reported changes in brain volumes, particularly in the frontal lobes, and prefrontal cortical thickness, using MRI morphometry in long-term meditators compared to non-meditators (Lazar et al., 2005; Hernández et al., 2016, 2020; Afonso et al., 2017). This is true for specific types of meditation as well as when meditators from different traditions are grouped together (Luders et al., 2012). Previously, our group demonstrated changes in brain function as assessed by cerebral blood flow in the midbrain, thalamus, putamen, caudate, prefrontal cortex, parietal cortex, in long-term meditators compared to non-meditators (Newberg et al., 2010a).

The present study explores the differences in resting cerebral glucose metabolism in long-term practitioners of a unique paired meditation method called Orgasmic Meditation (OM) compared to a group of non-meditating healthy controls. OM is a meditation practice that involves female clitoral stimulation with a partner. The practice lasts a specified 15 min with a few minutes of preparation prior to starting the practice and a brief concluding component after the practice. While this practice is called Orgasmic Meditation, the goal is not to achieve sexual orgasm, or climax, according to those performing the meditation. Rather the goal of the meditation practice is to use the sexual stimulation as a focus of meditation in order to achieve a sense of oneness with the partner and perhaps the larger universe. Of course, this raises an important question as to how similar the physiological effects of the practice are to sexual stimulation itself compared to more traditional forms of meditation.

In addition, while OM is a unique approach to meditation, there are many other types of practices that utilize various bodily processes and actions as a focus of meditation—for example there are forms of “walking” meditation and “breathing” meditation, as well as practices that focus on auditory, verbal, or visual stimuli. Thus, in OM, the goal is an intense meditative state achieved by using sexual stimulation as the focus of the practice. This is bolstered by subjective descriptions of the experience which do not use words related to sexuality or sexual arousal, but to feelings of connectedness, oneness, awareness, relaxation, and joy (Newberg et al., 2021).

As part of a study that measured the acute effects of the OM practice (previously published in Frontiers in Psychology; Newberg et al., 2021), we explored whether there were significant differences in resting cerebral glucose metabolism of OM practitioners when compared to non-meditating controls. The results could then be considered in comparison to those of other studies of long-term meditation practitioners to observe similarities and differences.

It is important to clarify that according to the OM practitioners, both the male and female participants are engaged in the meditation practice and there is a specific effect to the female as well as the male. A recent study of 125 pairs showed that performing the OM practice over time resulted in improved health measures such as an increased sense of closeness based on the Inclusion of Other in the Self scale (Prause et al., 2021). For these reasons, it seems of value to explore whether there are neurophysiological changes associated with the performance of the practice over longer periods of time. Further, since males and females would have both similarities and differences in terms of how the practice is performed and experienced, it would be helpful to determine how male and female participants compare in terms of long term changes in the brain.

For the present study, we used ^18^F fluorodeoxyglucose (FDG) PET to evaluate cerebral glucose metabolism at rest and compare the scans from OM practitioners to a group of non-meditating healthy controls. It should be noted that while the practice is performed in pairs, this analysis is based on the resting brain scans from individual practitioners. The resting scan is not dependent on the pairs or couples in which people perform the practice. Further, the practice can be performed with different individuals, and therefore, is not restricted to a specific pairing of two individuals or to individuals involved in a romantic relationship.

Based on the current literature, we hypothesized that there were several brain regions of particular interest that would be our focus. These regions included the basal ganglia associated with the reward network, limbic areas involved with emotional processing, frontal regions involved with focusing attention, and posterior regions involved with the default mode network. Each of these areas have been observed to be involved with meditation practices in other neuroimaging studies. For example, several studies by our group and others, have found altered frontal lobe function during meditation techniques that involve focused attention (Herzog et al., 1990; Newberg et al., 2001, 2010b). Studies of the long term effects of meditation have found thicker frontal cortices and more active frontal lobes at rest (Lazar et al., 2005; Luders and Kurth, 2019). We have also observed the parietal lobe to be affected during intense meditation practices particularly associated with a loss of the sense of self and feelings of self-transcendence (Newberg et al., 2001; Newberg and Iversen, 2003; Khalsa et al., 2009). Long term changes include increased activity in the prefrontal cortex, parietal cortex, thalamus, putamen, caudate, and midbrain in individuals performing these focused concentration techniques (Newberg et al., 2010a).

We would also hypothesize that the long term effects of OM would appear neurophysiologically similar to other meditation practices rather than simply sexual arousal or orgasm. Thus, there would likely be an effect of OM on cortical areas involved in higher thought processes rather than central structures such as the brain stem which is involved more with sexual function. We might expect increased metabolism in the limbic areas, dopamine areas, and thalamus in part due to the meditative component and in part due to sexual stimulation. We also hypothesized that there would be alterations in the structures of the default mode network including the parietal regions mentioned above, as well as the posterior cingulate cortex, since these have been involved in other groups of advanced meditators. Additionally, this is a paired practice, and therefore, we hypothesize that there might be changes in the social areas of the brain of both female and male participants, including the fusiform gyrus, angular gyrus, and supramarginal gyrus. Finally, we expected that there would be differences in the males and females as they are engaged in different components of the practice—one person providing the stimulation and the other receiving the stimulation. This should result in important differences in long term neurophysiological effects between the two types of participants in this practice.

## Methods

### Subjects

Subjects were recruited through the Department of Integrative Medicine and Nutritional Sciences at Thomas Jefferson University. All subjects provided written informed consent, approved along with the protocol by the Thomas Jefferson University Institutional Review Board. We studied 40 subjects in the OM group (M = 20; F = 20) which was based upon the effect size and standard deviations observed in our prior neuroimaging meditation studies (Newberg et al., 2010a). OM subjects were healthy individuals who were included if they performed the OM practice for over 1 year on a regular basis (2–3 times per week). We compared the OM group to 19 healthy non-meditating controls (M = 10; F = 9) who were included if they had no significant history of meditation practice (no ongoing practice and not tried any meditation more than 10 times in their life). Both OM and non-meditation subjects were excluded based on previous studies performed at our Department so that “(Luders and Kurth, 2019) they had any active psychological, physical, or neurological disorders that might affect cerebral metabolism (Afonso et al., 2020); they were taking medications that could alter cerebral blood flow or metabolism; and (Manna et al., 2010) they had significant claustrophobia or other reason why they could not lie still in the scanner. Female subjects were excluded if they were breastfeeding or pregnant (Newberg et al., 2023).”

### FDG PET imaging protocol

The FDG PET imaging was performed utilizing general standard of care procedures at the Marcus Institute of Integrative Health that have been previously reported (Newberg et al., 2023). All subjects initially had an intravenous catheter inserted with an attached intravenous saline bag placed on a moveable pole. After the subjects rested for approximately 10 min, 148 to 296 MBq of FDG were injected via a manual bolus. PET scans were acquired on a 3T Siemens mMR PET-MRI scanner (Siemens Medical Solutions USA, Inc., Malvern, PA) over approximately 30 min (Newberg et al., 2023).

### FDG-PET image processing

The ^18^F-FDG PET brain images were analyzed utilizing SPM12 (Wellcome Department of Cognitive Neurology, Institute of Neurology, London, UK) running on Matlab 2020b (MathWorks Inc., Sherborn, MA). The processing steps that were used for the analysis are provided as follows and were previously described (Newberg et al., 2023): “(1) Segmentation of the anatomical (T1-weighted) MRI data into gray matter (GM), white matter (WM), and cerebrospinal fluid (CSF) tissue compartments and creation of a skull-stripped version of the original anatomical scan used for PET-MRI co-registration. (2) PET-MRI co-registration: Using rigid-body transformations the spatial orientation of the PET and MRI images from the same subject were aligned. (3) Partial volume effect correction (PVEc) of PET images: voxel-based correction was applied using the Müller-Gärtner (MG) method provided by the PETPVE12 toolbox (Müller-Gärtner et al., 1992; Rousset et al., 1998). (4) Glucose Intensity normalization: The whole brain (gray and white matter) PET signal was used as a reference region to standardize the regional PET signal to standard uptake value ratio (SUVR) allowing for the direct inter-subject comparison of preprocessed PET data. (5) Normalization: deformations produced from the SPM12 segmentation implemented in step 1 were applied to the PVEc PET images to transform the functional data into the standard Montreal Neurological Image (MNI) space. (6) Smoothing: A 6-mm full-width at half maximum (FWHM) Gaussian kernel was used to smooth the final PVEc PET images.”

### Statistical analysis

Statistical analysis was performed using the statistical module in the Data Processing & Analysis for Brain Imaging (DPABI, V5.1_201201) (Yan et al., 2016) that was run on the MATLAB R2020b platform (The Math Works, Inc., Natick, MA, United States). For this study a voxel-wise two-sample *t-*tests was conducted to determine cerebral metabolic differences between the two groups—long term OM practitioners compared to the non-meditation control subjects. The resulting statistical maps were corrected for multiple comparisons to a voxel level significant value of *p* < 0.01 using the Gaussian Random Field (GRF) theory correction and a minimum cluster size of 25 voxels (2 × 2 × 2 mm per voxel) with the cluster level significant value set at *p* < 0.05). The clusters found to have significant group differences between the OM and non-meditation groups were corrected for multiple comparisons thus including only results that survived correction with the GRF approach. The regions are based on the Automated Anatomical Labeling (AAL) atlas with peak intensity values reflected in x, y, z coordinates of primary peak locations in the Montreal Neurological Institute (MNI) space.

## Results

The demographic information for the participants, including the mean age and meditation experience, is provided in Table 1. The breakdown by males and females is given below.

**Table 1:** Participant group demographic information.

**Variable**	**OM practitioners**		**Non-meditating controls**	
	**Females**	**Males**	**Females**	**Males**
	**(*****N** =* **20)**	**(*****N** =* **20)**	**(*****N** =* **9)**	**(*****N** =* **10)**
Mean age ± SD (years)	39.0 ± 10.1	40.8 ± 9.7	47.1 ± 13.9	42.8 ± 13.1
Age range (years)	28–66	28–65	23–61	21–59
Mean years of experience ± SD	7.8 ± 3.4	5.5 ± 1.9	—	—

There were no significant differences in age or gender between the meditation and control groups.

**Figure 1:**
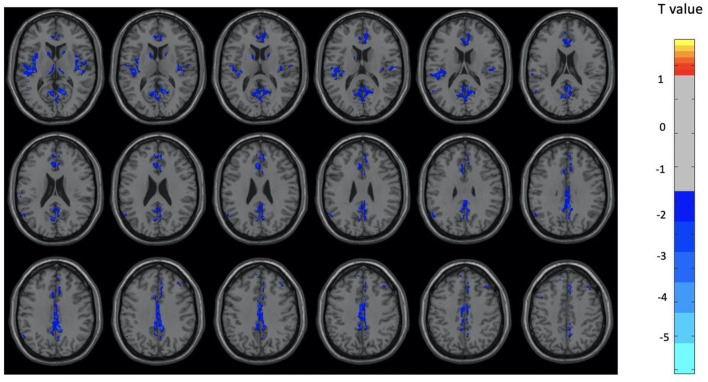
Brain regions with significant differences between the OM and control groups for the total subjects. Two-sample *t*-test with results presented for GRF-corrected *p* < 0.01 and a cluster size > 25 voxels.

**Figure 2:**
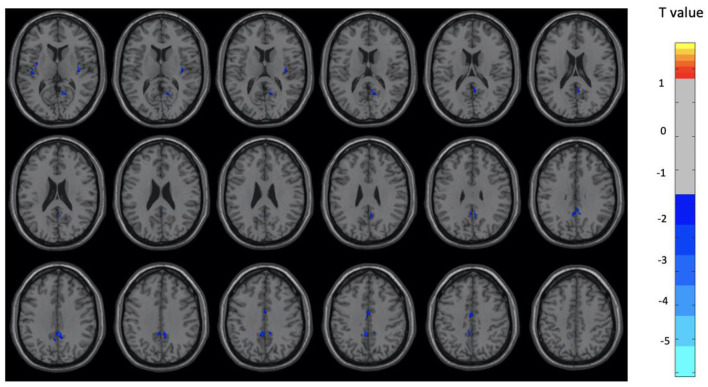
Brain regions with significant differences between the OM and control groups among male subjects. Two-sample *t-*test with results presented for GRF-corrected *p* < 0.01 and a cluster size > 25 voxels.

**Figure 3:**
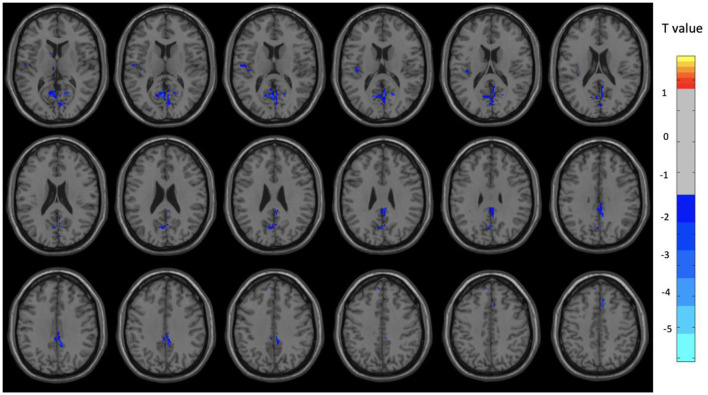
Brain regions with significant differences between the OM and control groups among female subjects. Two-sample *t*-test with results presented for GRF-corrected *p* < 0.01 and a cluster size > 25 voxels.

Overall, the OM group showed significant differences in metabolic activity at rest compared to the controls. Specifically, there was significantly lower metabolism in select areas of the frontal, temporal, and parietal lobes, as well as the anterior cingulate, insula, and thalamus, in the OM group compared to the controls. The structures that were significantly different based on two sample *t*-test analysis between the OM and non-meditation control groups are provided in Table 2 and Figure 1. The structures provided are those that were significant after Gaussian Random Field (GRF) theory correction for multiple comparisons with a cluster size >25 voxels. In addition, there were notable distinctions between the males and females with the females in particular demonstrating significantly lower metabolism in the thalamus and insula compared to female controls. Males demonstrated decreased metabolism in the cerebellum, left cingulate gyrus, left inferior temporal lobe, right middle temporal lobe, and left rectal gyrus. These results are provided in Tables 3, 4 as well as Figures 2, 3.

**Table 2:** Brain regions with significant differences between the OM and control groups for the total subjects.

**Structure**	**Peak coordinate**			**Peak intensity**	**Cluster size**
	**x**	**y**	**z**		**(voxels)**
Left Angular Gyrus (DMN)	−54	−60	28	−4.92	53
Left Inferior Parietal (FPN)	−46	−58	56	−4.93	26
Left Inferior Temporal	−56	−10	−32	−4.91	58
Left Middle Frontal	−30	4	52	−4.92	47
Left Middle Temporal (DAN)	−52	−10	−12	−4.91	224
Left Superior Medial Frontal	−6	48	44	−4.93	58
Left Temporal Pole	−46	12	−32	−4.93	29
Left Thalamus	0	−10	8	−4.92	80
Right Anterior Cingulate (VAN)	4	42	26	−4.91	29
Right Inferior Temporal	52	−4	−38	−4.93	90
Right Insula (VAN)	42	20	2	−4.91	675
Right Middle Frontal	38	34	36	−4.94	28
Right Middle Temporal (DAN)	54	0	−22	−4.92	143
Right Superior Frontal	30	24	56	−4.94	27
Right Temporal Pole	34	16	−32	−4.92	31

Two–sample *t*–test with results presented for GRF–corrected voxel–level *p* < 0.01, cluster–level *p* < 0.05, cluster size > 25 voxels. The regions are based on AAL atlas. Peak intensity values that are negative reflect decreased metabolism in the OM group. The x, y, z coordinates of primary peak locations are presented in the space of MNI.

**Table 3:** Male subjects, brain regions with significant differences between the OM and control groups.

**Structure**	**Peak coordinate**			**Peak intensity**	**Cluster size**
	**x**	**y**	**z**		**(voxels)**
Left Cerebellum	−44	−62	−40	−4.94	49
Left Cingulate Cortex	4	−44	42	−4.94	29
Left Heschl's Gyrus	−44	−18	10	−4.95	43
Left Inferior Temporal	−52	−38	−24	−4.94	57
Left Orbitofrontal Cortex (LN)	−18	26	−20	−4.94	102
Left Rectal Gyrus	−2	40	−18	−4.94	46
Right Cerebellum	6	−58	−42	−4.95	35
Right Fusiform	36	−24	−30	−4.95	65
Right Hippocampus (DMN)	26	−4	−22	−4.94	33
Right Middle Temporal (DAN)	52	−28	−6	−4.97	39
Right Orbitofrontal Cortex (LN)	18	20	−24	−4.93	71

Two-sample *t*-test with results presented for GRF-corrected voxel-level *p* < 0.01, cluster-level *p* < 0.05, cluster size > 25 voxels. The regions are based on AAL atlas. Peak intensity values that are negative reflect decreased metabolism in the OM group with x, y, z coordinates of primary peak locations in the space of MNI.

**Table 4:** Female subjects, brain regions with significant differences between the OM and control groups.

**Structure**	**Peak coordinate**			**Peak intensity**	**Cluster size**
	**x**	**y**	**z**		**(voxels)**
Left Fusiform	−22	−44	−18	−4.97	84
Left Heschl's Gyrus	−46	−20	10	−4.98	56
Left Insula (VAN)	−26	12	−18	−4.98	69
Left Middle Temporal (DAN)	−52	−36	−4	−4.99	34
Left Orbitofrontal Cortex (LN)	−24	24	−24	−4.99	34
Left Postcentral Gyrus (SMN)	−40	−32	64	−4.99	31
Left Precentral (SMN)	−38	2	54	−4.99	36
Left Precuneus (DMN)	−4	−54	8	−4.98	52
Left Superior Frontal	−20	30	58	−4.99	27
Left Thalamus	−6	−20	10	−4.98	73
Right Hippocampus (DMN)	22	−10	−12	−4.97	178
Right Inferior Temporal	48	−50	−18	−4.99	35
Right Insula (VAN)	38	−10	6	−4.99	205
Right Orbitofrontal Cortex (LN)	36	28	−20	−4.98	27
Right Superior Frontal	8	30	44	−5.02	40
Right Thalamus	12	−26	0	−4.98	51

Two-sample *t*-test with results presented for GRF-corrected voxel-level *p* < 0.01, cluster–level *p* < 0.05, cluster size > 25 voxels. Peak intensity values that are negative reflect decreased metabolism in the OM group. The x, y, z coordinates of primary peak locations are in the space of MNI.

The individual structures observed in the tables below are also part of seven major brain networks typically identified by the Yeo atlas (Yeo et al., 2011) which include the Visual network (VIN), Somatomotor network (SMN), Dorsal attention network (DAN), Salience network (SAN), Limbic network (LIN), Frontoparietal network (FPN), and Default mode network (DMN). Structures specifically associated with these networks are annotated in the tables.

## Discussion

The above results represent the first study that has utilized FDG PET to evaluate the long term neurophysiological effects of a meditation practice, and specifically a practice that involves a focus on clitoral stimulation as a paired practice between two individuals. This stimulation is used as the focus of the meditation. While there is a person who provides the sexual stimulation and another who receives it, both individuals are regarded as engaging in the meditative practice. And while both participants describe the practice as a meditative state, because they are involved in distinct ways, it is also expected that there would be different physiological effects, both during the practice itself and on the individuals over time. It should be noted that although a female is always receiving the stimulation, the other participant can potentially be male or female. However, for consistency in our study, we had half males and half females.

This report builds on our prior publication of the acute effects of the OM practice itself. We hypothesized that long term changes in resting brain function in individual OM practitioners would occur in brain structures that have been shown to be affected during OM, as well as other meditative practices, but could also have some similarities to sexual stimulation as well. We report here evidence that there are resting effects from doing the OM practice for prolonged periods of time. Thus, our results are consistent with our general hypotheses.

Overall, when the entire group was compared between OM and non-meditation subjects, there were a number of brain areas that were significantly different, and particularly with lower metabolism in the OM group. For the entire OM group, compared to non-meditation subjects, there was significantly lower metabolism in the angular gyrus, inferior parietal, inferior temporal, middle frontal, insula, and thalamus. As will be discussed below, these structures are part of several important neural networks in the brain that have been shown to be involved in a variety of other meditation practices.

It is interesting that there were overall reductions in metabolism in these brain regions as this would suggest a consolidation of brain responses to the prolonged and repeated engagement in the practice. On the other hand, several meditation studies, including our own, have found overall higher activity in long term meditators in several similar regions, particularly the frontal lobes. We previously reported higher cerebral blood flow in long term meditators of focused concentration based practices in the prefrontal cortex, parietal cortex, thalamus, putamen, caudate, and midbrain (Newberg et al., 2010a). Studies of brain morphometry found increased brain volumes, particularly in the frontal cortices in advanced meditators. Areas that were particularly significantly increased included the frontal lobe (particularly the prefrontal cortex) and insula (Luders et al., 2012).

However, most of these practices have employed intense concentration or focused attention on a cognitive process, which is not a primary element of OM. In fact, much of the OM practice is associated with receptive experiences (receiving sensory or social cues) which are more likely associated with decreases in complex cognitive networks. Furthermore, the overall experience of this meditation is initially associated with arousal, but is then followed by significant calmness suggesting an overall reduction in brain activity.

MRI studies of functional connectivity in long term meditators have indicated areas of both increased and decreased connectivity compared to non-meditating controls. It should be noted that cerebral glucose metabolism appears to generally correlate with functional connectivity. Thus, areas of increased connectivity should be associated with higher metabolism and areas of decreased connectivity should be associated with lower metabolism (Tomasi et al., 2013; Shokri-Kojori et al., 2019). Hence, the reduced metabolism observed in OM subjects suggests an overall reduction in resting functional connectivity, although this has yet to be evaluated. How might such findings compare to studies of functional connectivity in other types of long term meditators?

Taylor et al. (2013) found that the functional connectivity between the right inferior parietal lobe and dorsal medial PFC was higher in experienced meditators compared to novices suggesting that long-term meditation may improve global attention. A study of 16 experienced meditators demonstrated decreased functional connectivity between the dorsal attention network (includes structures such as the intraparietal sulcus, middle temporal region, frontal eye fields, and precentral gyrus) and DMN (includes the posterior cingulate, medial PFC, superior temporal region, and angular gyrus) (Devaney et al., 2021).

The DMN has been observed to be particularly affected by meditation practices due to their prominent focused attention element since the DMN is active at rest and becomes less active while performing concentration tasks such as meditation. The DMN refers to a number of separate brain structures, including the medial prefrontal cortex, and inferolateral temporal cortex, precuneus, inferior parietal lobule, and posterior cingulate cortex, that work in synchrony when the brain is at rest (Raichle et al., 2001). Brewer et al. (2011) explored the effect of several different meditation types on the DMN, showing that the main nodes of the DMN were deactivated in experienced meditators. The authors also found a strong physiological coupling between regions involved in cognitive control and self-monitoring during meditation, specifically the dorsal anterior cingulate, dorsolateral prefrontal regions, and posterior cingulate cortex. Other studies of long term meditation have similarly found changes in the DMN (Jang et al., 2011). However, other brain networks are also affected by long term meditation practice including the salience network, somatomotor network, limbic network, and dorsal attention network (Hasenkamp and Barsalou, 2012; Shao et al., 2016; Cotier et al., 2017; Barrós-Loscertales et al., 2021; De Filippi et al., 2022; Kral et al., 2022). Many of these areas were also found to be significantly different in the current subjects who have engaged in the OM practice for a prolonged period of time.

In spite of the complex and mixed findings when considering all of the studies on the long term neurophysiological effects of meditation, none of them explored a practice that directly resembles the unique characteristics of the OM practice. In our current study, we found regions such as the temporal lobe, precuneus, cingulate cortex, inferior parietal lobe, and hippocampus, that are involved with the DMN, were significantly reduced in OM practitioners compared to non-meditating controls. Structures of the salience network were also affected by OM including structures such as the insula, anterior cingulate, and thalamus. And as above, these areas have been implicated in other studies of brain changes associated with other meditation practice over long periods of time.

There are only a few studies that have explored cerebral metabolic changes associated with the long term practice of meditation. A small study of 10 Ashtanga yoga practitioners with an average of 5 years of experience revealed decreased resting regional glucose metabolism in the medial temporal cortex, striatum, and brainstem compared to controls (van Aalst et al., 2020). These findings are similar to the current study which showed decreases in the temporal areas, although not in the striatum or brain stem.

In addition to the general findings of decreased metabolism in long term OM practitioners compared to non-meditation subjects, there were distinctions between the significant findings in males and females in our study group. There were similar findings of lower resting metabolism in both males and females in the fusiform gyrus, Heschl's gyrus, orbitofrontal cortex, and hippocampus. These are regions that have been described to be associated with long term meditation effects as described above and have also been associated with various social orienting processes, an element related to OM as it involves two individuals performing the practice together.

Males had uniquely lower resting metabolism compared to controls in the cerebellum, cingulate gyrus, fusiform gyrus, left inferior temporal and right middle temporal regions. Females had uniquely lower resting metabolism compared to controls in the thalami, insula, superior frontal, left precuneus, left middle temporal, and right inferior temporal regions. These distinctions are consistent with the hypothesis that there should be differences between males and females engaged in the OM practice. Further, the areas involved in males are primarily related to social and memory processes as well as regulation of emotions. The cerebellum is likely more associated with the motor activity involved with the individual providing manual stimulation that has to be well coordinated with the partner as part of the practice.

The areas affected in females are also involved in social and memory processes, but includes the thalamus and insula which are particularly involved in sexual stimulation as well (Georgiadis et al., 2006). Interestingly, studies of sexual climax have generally been associated with decreases in cerebral blood flow in the cortex when compared with the control condition, particularly involving the left lateral orbitofrontal cortex, anterior temporal pole, and inferior temporal gyrus (Georgiadis et al., 2006). During the OM practice, climax is not specifically achieved, but it might be that repeated sexual stimulation leads to reductions in metabolism in brain areas associated with sexual climax. Temporal lobe and frontal lobe activity has been shown to decrease after ejaculation in men, although the middle temporal gyrus and orbitofrontal cortex activity was found to increase (Tiihonen et al., 1994; Georgiadis et al., 2006). Frontal lobe activation has also been observed in women during orgasm, and decreased activity in the frontal lobe regions were reported in men based on PET scan and perfusion fMRI data (Komisaruk and Whipple, 2005).

While there are few studies of prolonged frequent sexual experience on the baseline function of the human brain, animal studies have suggested that sexual experience modifies activity in the striatum (Bradley et al., 2005), again, an area that was not specifically affected in the long term OM practitioners. Further, problematic hypersexual behavior has also been associated with increased reactivity in the striatum and changes in connectivity between the superior temporal region and striatum, areas not found to be affected by OM (Gola and Draps, 2018; Seok and Sohn, 2018). Thus, the findings of the present study seem more related to long term effects of meditation rather than sexual stimulation and related behaviors.

Future research can potentially explore more specific neurophysiologic correlates in long term OM practitioners, and perhaps use other comparison groups. It is also important for future studies to consider the gender bias in the present study because we had only males-female pairs and there can also be female-female pairs. Future studies should involve a larger sample size which would also help confirm various correlations between length of practice, the approximate number of times performing the practice, as well as various clinical and demographic variables.

The present results demonstrate that the OM practice involves a number of brain regions that are also affected in other groups of long term meditators. However, OM also has unique neurophysiological changes that distinguish it from other meditative practices. Many of the brain changes observed in the present study share similarities with those observed in studies of concentrative meditation practices as well as mindfulness meditation. Specifically, we found that long term practitioners of OM have changes in the resting metabolism of the frontal lobe and structures of the default mode network. Other brain changes are similar to those involved with sexual stimulation. However, the specific pattern of cerebral metabolism observed in both male of female experienced OM practitioners indicates that it is a unique practice that has long term effects on its participants. Such functional neuroimaging findings might have implications for how OM could be used to modify brain activity in various conditions.

While there are the typical challenges and limitations with performing neuroimaging studies in terms of accuracy, specificity, and reproducibility of findings, when evaluating meditation practices, there are several important and challenging limitations of these studies. One limitation has to do with selecting the appropriate comparison group. It could be argued that in addition to a non-meditating control, we could consider adding a group of individuals who performed the same type of sexual stimulation, but without meditating. This is problematic for several reasons. First, sexual stimulation of this type would be considered an active process, and thus, the comparison would not be against a normal baseline but a group doing another type of practice. In fact, it might be argued that future studies should compare OM to other types of meditation practices. This would certainly be of benefit for determining which practice produces the greatest results or would be more likely to produce a clinical effect. For example, it might be important to differentiate the benefits of OM to mindfulness in a population of patients with depression or anxiety. The other problem with using a group that undergoes frequent sexual stimulation similar to OM is that these individuals would have to do this type of specific sexual stimulation 2–3 times per week over a year or more. This seems unlikely to find such a population naturally occurring, or to create one as part of the study.

It should be noted that most meditation studies have a similar limitation regarding the appropriate selection of comparison groups, especially when natural bodily processes are used as a focus. For example, if one were to study a “walking meditation” it is not clear what would be the appropriate comparison—a non-walking but movement oriented practice (e.g., yoga or tai chi) a non-walking meditation (and if so, which one), a walking group that is not meditating, or perhaps a group involved in walking as exercise. In addition, people walk as part of their natural living, just as people engage in sexual activity throughout their life. In this case, it becomes challenging to determine whether and how to account for these naturally occurring activities if one is to create a comparison group that will match the target meditation group. In other words, how much additional walking would be needed and how is that determined based on how much baseline walking a given person does in their usual daily activities. It is always a challenge to determine the most appropriate comparison groups and how to incorporate them into a study of meditation techniques.

Another important limitation in long term meditation studies is not with respect to the imaging technique, but in the method used to determine what is meant by “long-term” when it comes to meditators. The obvious overall goal is to evaluate people who have extensive meditation experience. But this can be defined and evaluated in a number of ways. Perhaps the most important problem is that most reports of experience are based on self-reporting, as in the current study, in which subjects are asked about the amount and duration of their experience. This requires a degree of reporting accuracy. However, it may be difficult for anyone to accurately assess how much meditation they have done in the past. More importantly, it is unclear whether the number of times, the length of time, gaps in practice, the frequency, or the intensity is the most relevant. All of these are potentially important questions for not only understanding how to interpret neuroimaging results, but also in terms of what recommendations should be made for those interested in pursuing meditation for therapeutic purposes.

Future neuroimaging studies can address the limitations in the present preliminary study, and can help to corroborate and expand on the findings related to the long term effects of the OM practice. Such work can have important implications for the ongoing study of the long term effects of meditation practices in general, and OM in particular, as well as help assess potential outcomes in clinical trials.

## Data availability statement

The raw data supporting the conclusions of this article will be made available by the authors, without undue reservation.

## Ethics statement

The studies involving humans were approved by the Thomas Jefferson University IRB. The studies were conducted in accordance with the local legislation and institutional requirements. The participants provided their written informed consent to participate in this study.

## Author contributions

AN: Conceptualization, Data curation, Funding acquisition, Investigation, Methodology, Project administration, Resources, Supervision, Writing – original draft, Writing – review & editing. NW: Conceptualization, Data curation, Investigation, Methodology, Project administration, Supervision, Writing – original draft, Writing – review & editing. CH: Data curation, Investigation, Methodology, Project administration, Writing – original draft, Writing – review & editing. FV: Conceptualization, Formal analysis, Methodology, Software, Writing – original draft, Writing – review & editing. SG: Conceptualization, Formal analysis, Investigation, Methodology, Writing – original draft, Writing – review & editing. RR: Conceptualization, Data curation, Formal analysis, Investigation, Methodology, Supervision, Writing – original draft, Writing – review & editing.
